# Identification of World War II bone remains found in Ukraine using classical anthropological and mitochondrial DNA results

**DOI:** 10.1007/s00414-019-02026-z

**Published:** 2019-03-13

**Authors:** Eszter Dudás, Éva Susa, Horolma Pamjav, Zoltán Szabolcsi

**Affiliations:** 1 Department of Reference Samples Analysis, Hungarian Institute for Forensic Sciences, Institute of Forensic Genetics, Gyorskocsi u. 25, Budapest, H-1027 Hungary; 2 Anthropologist, Institute of National Heritage, Fiumei str. 16-18, Budapest, 1086 Hungary; 3 Georgikon Faculty, Department of Animal Sciences and Animal Husbandry, University of Pannonia, Deák Ferenc u. 16, Keszthely, H-8361 Hungary

**Keywords:** Forensic science, Forensic genetics, Mitochondrial DNA, Forensic anthropology, Second World War, Identification

## Abstract

Gyula Ágner was a Royal Hungarian First Lieutenant (1st Lt.) during the World War II and died at 30 years old due to a mine shrapnel injury on 27 April 1944 in Luczky, Ukraine. In October 2014, the Hungarian Ministry of Defence exhumated the remains then transported them to Budapest in Hungary. Classical anthropological methods were used to determine morphological gender, height and age at death; furthermore, metrical and pathological characters were also analysed. Determination of maternal lineage was the only solution to examine the possible relationship of the bone fragments. Gyula Ágner did not have direct descendants, thus the living niece of the deceased (his sister’s daughter) served as the reference person during the investigations. Hypervariable regions of the mtDNA control region (HV1, HV2 and HV3) were amplified by Qiagen® Multiplex PCR Kit in different monoplex reactions. The results of the anthropological and genetical analysis supported the hypothesis that the bone remains belong to Gyula Ágner.

**Electronic supplementary material:**

The online version of this article (10.1007/s00414-019-02026-z) contains supplementary material, which is available to authorized users.

## Introduction

Gyula Ágner was born on 31 May 1914 in Budapest, Hungary [Supplementary Material, Fig. S1]. After graduating from high school, he joined the Hungarian Royal Army. He died a heroic death on 27 April 1944 due to a mine shrapnel injury close to Luczky, Ukraine, where his body was buried in the churchyard. Gyula Ágner received the highest Hungarian military honour, the *Hungarian officer’s gold medal for bravery.* In 2014, the Hungarian Ministry of Defence made an investigation in Luczky, Ukraine, and they found the putative remains of Gyula Ágner. The former Institute of Forensic Medicine, Network of Forensic Science Institutes (now Hungarian Institute for Forensic Sciences) made several identifications before [1, 2], therefore the Hungarian Ministry of Defence asked the Institute to identify of the bone remains. Anthropological and mitochondrial DNA analyses were then made on the remains. First Lieutenant Gyula Ágner did not have any direct descendants. The reference DNA sample (buccal swab) was collected from Gyula Ágner’s living niece (his sister’s daughter) to examine the maternal relatedness.

## Materials and methods

### Anthropological methods

First, we separated, cleaned and ordered the bones anatomically (Supplementary Material, Fig. S2). Afterwards, we determined the biological profile. We carried out sex determination based on Éry et al. [3], age at death based on Meindl and Lovejoy [4] and Nemeskéri et al. [5], furthermore, height based on Sjøvold [6] and craniometrical analysis based on Martin and Saller [7] methods. We recorded the dental status using Ubelaker’s [8] and Huszár’s [9] method.

**Fig. 1:**
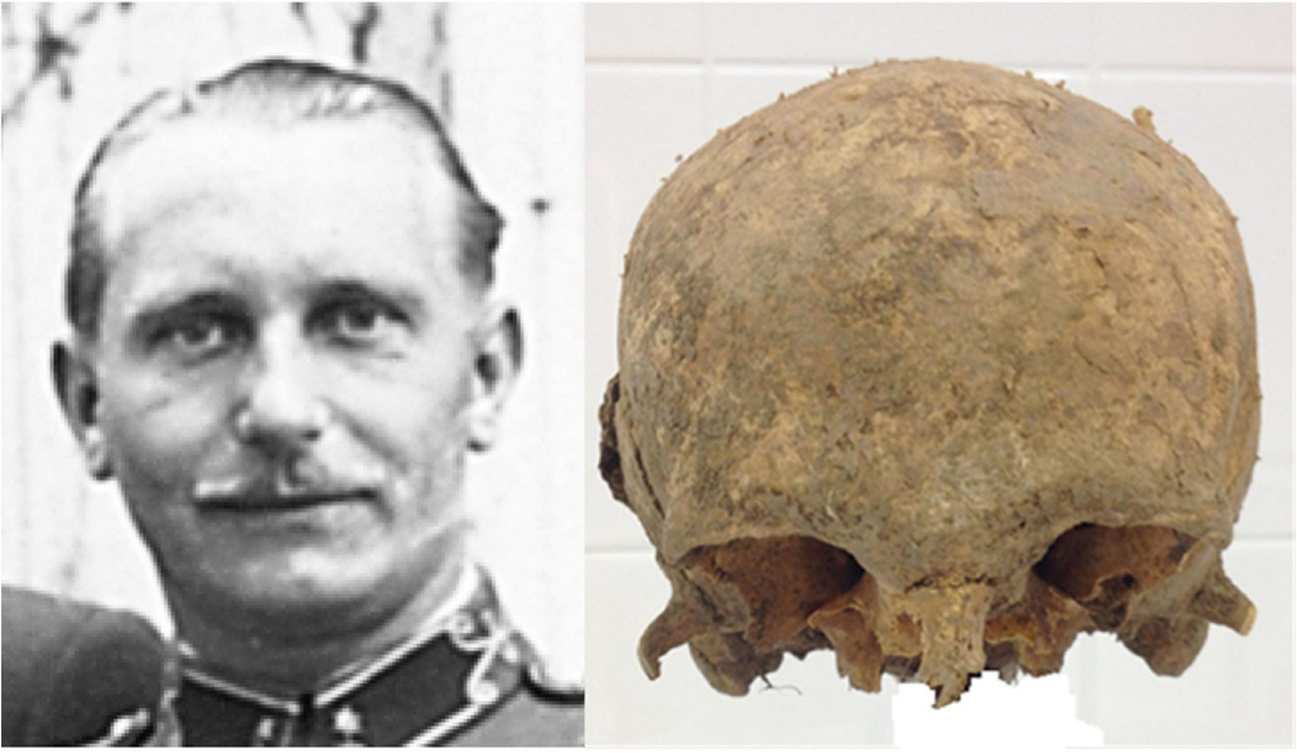
The comparative morphological analysis between a photo of the late Gyula Ágner and the skull

Fortunately, there were period photos of the late Gyula Ágner so we could do a comparative morphological analysis between the photos and the skull (Fig. 1).

### Genetic methods

#### Sampling and DNA isolation

We used a DNA extraction method of bone samples developed by us. We cut the bone sample from the diaphysis of the leftward femur (Supplementary Material, Fig. S3). The cut particle of the bone was cleaned with dental polisher. The polished particle was decontaminated one time for 2 min using the chemical (alkaline-hypochlorite, 14 g/l) impact. The remains of the chemical material were removed from the surface of the particle by using water wash three times and once with 96% ethanol flush. Then the particle was grinded and powdered with Retsch® MM 200 Mill. DNA was isolated from 350 mg bone powder using EZ1 DNA Investigator Kit and EZ1 Advanced Instrument (Qiagen®). The buccal swab of the reference sample was collected with Whatman EasiCollect® and DNA was isolated using EZ1 DNA Investigator Kit and EZ1 Advanced robot system (Qiagen®).

#### PCR amplification and sequencing

The mtDNA hypervariable regions (HV1, HV2 and HV3) were amplified in different monoplex reactions using Qiagen® Multiplex PCR Kit. For BigDye sequencing PCR, F15971/R16401 primers for the HV1, the L48/H408 primers for HV2 and the F403/R599 primers for the HV3 were used. To sequence the fragments, we used an ABI 3130 capillary electrophoresis instrument. The following regions of mtDNA hypervariable regions were sequenced: HV1, 16024–16,375; HV2, 70–375; HV3, 451–546. To align the sequences, we used Sequencing Analysis v.5.4 and SeqScape v2.7 software and the sequences compared to the revised Cambridge Reference Sequence (rCRS, GenBank: NC_012920) [10]. We followed the ISFG recommendations for the haplotype nomenclature [11].

## Results and discussion

### Anthropological results and discussion

Based on the investigation, the remains belonged to an approx. 30–35 years old male who was approx. 175 cm tall. There were many dental treatments (inlays, crowns and bridges) during his life but the abrasion of the teeth belonged to the *abrasio superficialis I* group. This means that based on the dental status, the remains belonged to a young person (approx. 30 years). We did not find any pre- and perimortem injuries on the bones. All the injuries were postmortem. The comparative morphological analysis between the period photos and the skull was limited because most of the face skull was damaged but those details which were suitable for the analysis showed similar characteristics with the photos. These similar characteristics were:

- broad and high forehead,
- expressed *glabella* and *arcus superciliaris*,
- expressed *processus zygomaticus ossis frontalis* and
- broad nasal bridge.

Based on our results, the anthropological features did not exclude the possibility that the bone remains were those of 1st Lt. Gyula Ágner.

### Genetic results and discussion

Based on the results of the mtDNA sequencing, we repeatedly detected the same haplotype between the bone sample and the living reference person. The detected mtDNA haplotype can be found in Table 1.

**Table 1:** mtDNA sequence differences from the rCRS sequence in the reference and the bone samples. Sequenced regions: HV1, 16024–16,375; HV2, 70–375; HV3, 451–546

Samples	mtDNA sequence differences from the rCRS sequence			
Reference person	A263G	− 315.1C	− 315.2C	T16093C
Bone sample	A263G	− 315.1C	− 315.2C	T16093C

The detected haplotype belongs to haplogroup H based on EMPOP search [12]. The HV1-HV3 haplotype did not give any match among metapopulations of the EMPOP database according to the search on 4 January 2019. The unbiased estimation of the frequency of the haplotype in the European population reached the value at 5.06 × 10^−4^ using confidence limit from zero proportion [13]. The detected haplotype can be considered as a very rare one both in European and worldwide context. According to the genetic result, it is highly supportable that is the two samples/persons are closely related within the maternal lineage.

## Conclusion

Both the anthropological and genetic results support the hypothesis that the bone remains, which were exhumed from the putative grave of Gyula Ágner, really belong to 1st Lt. Gyula Ágner who died a heroic death on 27 April 1944. The reburial was carried out with military honours on 12 of May 2016 in Fiume Road National Graveyard, Budapest, Hungary.

## Electronic supplementary material

ESM 1 (JPG 131 kb)

ESM 2 (DOCX 564 kb)

ESM 3 (DOCX 249 kb)
