# Supplementary figures and images for: Identification of World War II bone remains found in Ukraine using classical anthropological and mitochondrial DNA results
Source: Int J Legal Med. 2019 Mar 13;134(2):487–9. doi: 10.1007/s00414-019-02026-z (PMC7044253; doi:10.1007/s00414-019-02026-z)

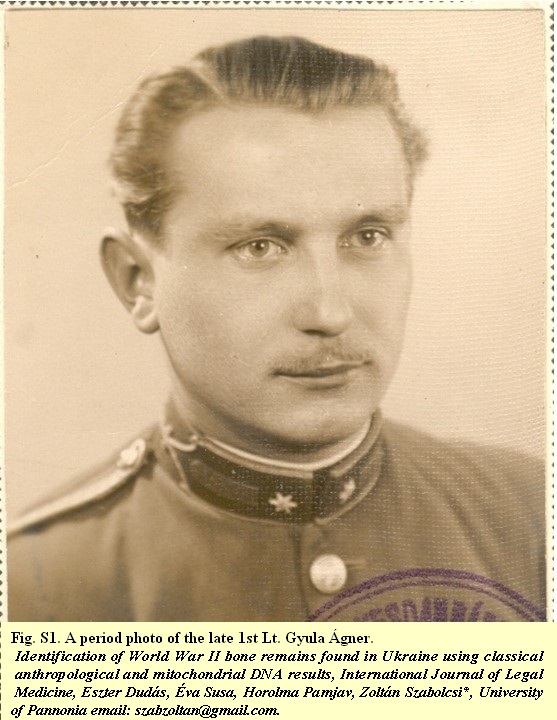

Supplement: Supplementary file 1 — (JPG 131 kb) [file 414_2019_2026_MOESM1_ESM.jpg]
