# Supplementary material for: Identification of World War II bone remains found in Ukraine using classical anthropological and mitochondrial DNA results
Source: Int J Legal Med. 2019 Mar 13;134(2):487–9. doi: 10.1007/s00414-019-02026-z (PMC7044253; doi:10.1007/s00414-019-02026-z)
Supplement: Supplementary file 3 — (DOCX 249 kb) [file 414_2019_2026_MOESM3_ESM.docx]

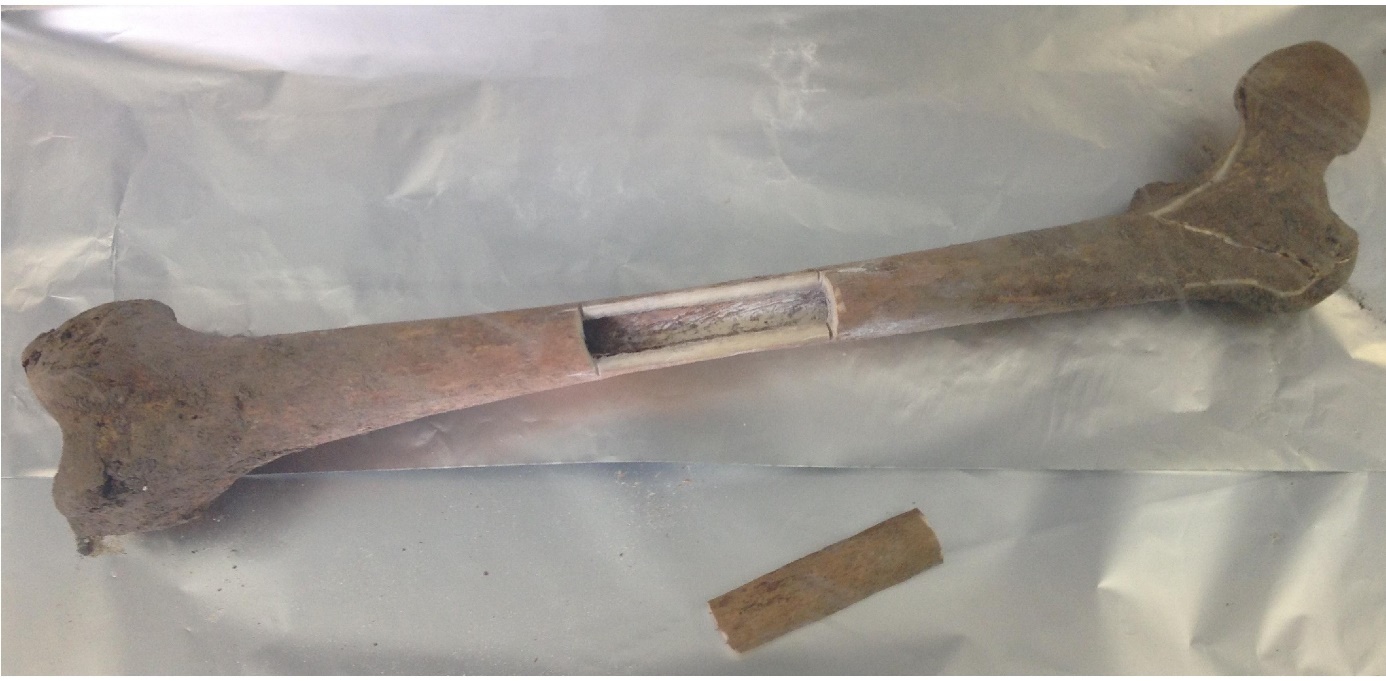


Fig. S3. The post examination leftward femur.

**Identification of World War II bone remains found in Ukraine using classical anthropological and mitochondrial DNA results,** Eszter Dudás, Éva Susa, Horolma Pamjav, Zoltán Szabolcsi^*^**, University of Pannonia, email: szabzoltan@gmail.com**
